# The circadian clock and metabolic homeostasis: entangled networks

**DOI:** 10.1007/s00018-021-03800-2

**Published:** 2021-03-08

**Authors:** Leonardo Vinícius Monteiro de Assis, Henrik Oster

**Affiliations:** Center of Brain, Behavior and Metabolism, University of Lübeck, Institute of Neurobiology, Marie Curie Street, 23562 Lübeck, Germany

**Keywords:** Circadian rhythms, Clock genes, Tissue clocks, Energy metabolism, SCN

## Abstract

The circadian clock exerts an important role in systemic homeostasis as it acts a keeper of time for the organism. The synchrony between the daily challenges imposed by the environment needs to be aligned with biological processes and with the internal circadian clock. In this review, it is provided an in-depth view of the molecular functioning of the circadian molecular clock, how this system is organized, and how central and peripheral clocks communicate with each other. In this sense, we provide an overview of the neuro-hormonal factors controlled by the central clock and how they affect peripheral tissues. We also evaluate signals released by peripheral organs and their effects in the central clock and other brain areas. Additionally, we evaluate a possible communication between peripheral tissues as a novel layer of circadian organization by reviewing recent studies in the literature. In the last section, we analyze how the circadian clock can modulate intracellular and tissue-dependent processes of metabolic organs. Taken altogether, the goal of this review is to provide a systemic and integrative view of the molecular clock function and organization with an emphasis in metabolic tissues.

## Function and organization of the circadian timing system

### How organisms keep track of time

The rotation of the Earth around its own axis creates a precise 24-h natural light and dark rhythm. This predictability promoted the evolution of internal timekeeping systems that serve to anticipate and adapt to associated changes in environmental demands. Indeed, such internal *circadian* clock systems can be found from uni to multicellular organisms with an increasing complexity in terms of mechanism and organization [1, 2].

Mammals possess a hierarchical circadian system with a central oscillator, the suprachiasmatic nucleus (SCN), localized in the hypothalamus [3]. The primary role of SCN is to provide subordinate clocks in the brain and peripheral tissues, through several pathways (See "Overview of SCN-driven inputs to peripherel clocks"), with temporal information aligning a multitude of clock-associated biological processes to a single time zone shared by the entire organism in line with the cyclic demands posed by the environment [4–9]. Disbalance or disruption of this temporal harmony has been shown to contribute to the development and progression of several diseases [2, 5, 10, 11].

The main synchronizing (or *entraining*) signal of the circadian clock is light. In the early 2000s, pioneering studies by Ignacio Provencio demonstrated the presence of a novel non-visual photoreceptor in the mammalian retina, melanopsin (OPN4) [12, 13]. OPN4 was shown to participate, together with cone and rod photoreceptors, in the synchronization of the SCN to the environmental light–dark cycle [14, 15]. Two isoforms of OPN4 are expressed in a subset of intrinsically photosensitive retinal ganglion cells (ipRGCs) [12, 16, 17] and are further subdivided into five categories [16–18]. OPN4 not only serves as a regulator of circadian rhythms, but it also controls several other light-responsive biological processes such as pupil constriction, melatonin synthesis, glaucoma development, migraine photophobia, and sleep [19]. Upon photon capture by ipRGCs, light information is transformed into electric pulses that travel through the retinohypothalamic tract (RHT), a monosynaptic pathway that innervates the ventromedial (or *core*) portion of the SCN containing vasoactive intestinal polypeptide (VIP)-expressing neurons. Glutamate and pituitary adenylate cyclase-activating polypeptide (PACAP) are released from ipRGC termini in the SCN (Reviewed in [20]). As the core SCN receives direct input from the retina, its photosensitivity leads to a fast increase in transcription of some genes, including the *Period* (*Per1/2*) clock genes (*Pers*), and increased neuronal firing. Single-cell transcriptomics reveal eight cellular subtypes within the SCN, of which five are neurons [21]. There exists intensive communication between the SCN’s core and its dorsoventral (arginine vasopressin-expressing) *shell* region. The latter has an essential role as the central circadian oscillator, which is beyond the scope of this paper, but the reader is referred to a recent review [22]. Glutamate-induced upregulation of *Per* gene expression is mainly depended on cAMP-CREB signaling during the night, which results in phase resetting of the molecular circadian clock machinery in the SCN pacemaker [20].

**Fig. 1:**
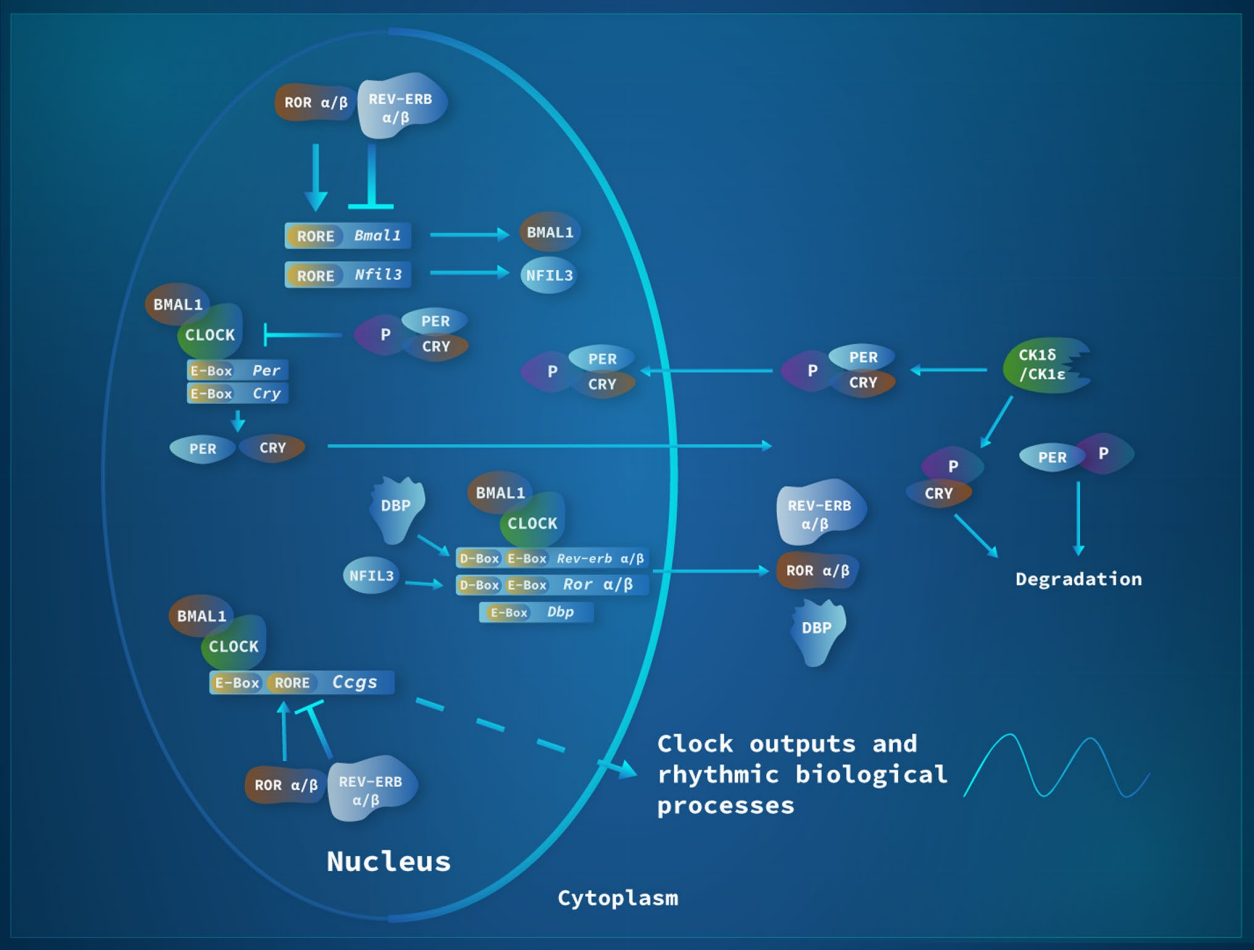
The molecular mechanism of the circadian clock in mammals. Every 24 h, a complex machinery comprised of genes and proteins undergoes changes in mRNA and protein levels through interlocked transcriptional-translational feedback loops (TTFLs). The reader is referred to the text for a detailed description of this mechanism. Abbreviation of the genes: *Per* = *Period*; *Cry* = *Cryptochrome*; *Clock* = *Circadian Locomotor Output Cycles Kaput*; *Bmal1* = *Brain and Muscle ARNT-Like 1*; *CKδ* = *Casein Kinase 1δ*; *CK1ε* = *Casein Kinase 1ε*; *Nfil3* = *Nuclear Factor, Interleukin‑3 Regulated*; *Rev-erbα/β* = *Nuclear Receptor Subfamily 1 group D Member 1 and 2*; *Rorα/β* = *Nuclear Receptor Subfamily 1 Group F Member 1/2*; *Dbp* = *Albumin D‑site Binding Protein*

At the cellular level, mammalian circadian clocks are based on interlocked transcriptional-translational feedback loops (TTFLs) comprised of positive and negative arms. In the beginning of the subjective day (circadian dawn), expression of *Per* (*1–3*) and *Cryptochrome* (*Cry1/2*) genes starts through direct action of the basic helix-loop-helix (bHLH) transcription factor heterodimer, Circadian Locomotor Output Cycles Kaput (CLOCK), or its paralogue Neuronal PAS Domain Protein 2 (NPAS2), and Brain and Muscle ARNT-Like 1 (BMAL1 or ARNTL), acting on *E-box cis*-regulatory enhancer regions. As gene transcription increases throughout the day, PER/CRY heterodimers accumulate, first in the cytoplasm and then in the nucleus, to represses the activity of CLOCK/BMAL1, leading to a reduction of *Per* and *Cry* gene transcription during the subjective night. Critically, PER and CRY protein stability is dependent on post-translational modifications that initiate protein degradation. Casein Kinase 1 δ and ε are responsible for PER protein phosphorylation and degradation while CRY stability is regulated by F-box and Leucine Rich Repeat Protein 3 (FBXL3)-mediated ubiquitination. Once negative-feedback repression is relieved through PER/CRY protein degradation, a new circadian cycle is initiated. In addition to this main TTFL, a secondary loop involves CLOCK/BMAL1 activating the expression of *Nuclear Receptor Subfamily 1 group D Member 1 and 2* (*Nr1d1/2*, also known as *Rev-erbα/β*) and *nuclear receptor Subfamily 1 group F Member 1, 2, and 3* (*Nr1f1-3*, also known as *Rorα*-γ). Through competition for binding to orphan receptor response elements (*ROREs*) in the *Bmal1* promoter, REV-ERBs and RORs regulate rhythmic *Bmal1* transcription. A third loop consists of BMAL1/CLOCK driving the expression of the PAR-bZip (proline and acidic amino acid-rich basic leucine zipper) transcription factor DBP (Albumin *D‑site* Binding Protein) that interacts at *D-box* enhancer regions with the repressor NFIL3 (Nuclear Factor, Interleukin‑3 Regulated, also known as E4BP4), expression of which is driven through the REV-ERB/ROR loop. Such *D-box* elements are found in the *Per* promoter regions (Fig. 1).

Cyclic chromatin modifications contribute to the stability of the TTFL system and are associated with circadian gene transcription [23]. Through the above-mentioned circadian enhancer motifs hundreds to thousands of tissue-specific clock-controlled genes (*CCGs*) are regulated in a circadian fashion by the core clock machinery [24]. Collectively, the resulting rhythms of gene/protein abundance allows the organism to keep track of time at the molecular level (reviewed in [9, 22, 25–27]). Of note, recent advancements in proteomics suggest that there is only a moderate degree of overlap between rhythmic transcripts and their respective protein products, which argues for a major function of post-transcriptional and translation modifications in circadian timekeeping (reviewed in [28]). As just one example, RNA methylation by N6-adenosine Methyltransferase 70 (METTL3) was shown to affect the period of the molecular clockwork in mice [29].

The molecular clock machinery is not restricted to the SCN. It is, at least in mammals, rather expressed in most—if not all—cells and tissues of the body, thus forming a systemic network of cellular clocks. One may stress that molecular rhythms based on TTFLs are less pronounced or absent in stem cells, but they become detectable as cells differentiate (reviewed in [30]). Several key genes that encode important regulators of biological processes are directly or indirectly affected by the clock gene machinery, a mechanism that ultimately allows biological processes to be within the same time zone [25, 27]. In mice, more than 40% of all protein coding genes display rhythmic transcription in at least one tissue of the body [24].

### Organization of the circadian clock network

The most accepted system of circadian organization is the “Orchestra Model”—a hierarchical system. In this orchestra, the SCN is considered the maestro, responsible for providing complete information and ensuring coordination among the different band members, i.e., the peripheral clocks. Each musician (tissue/organ) can play its own instrument (regulate biological processes), but the coordination is ensured by the maestro (SCN) [31, 32]. Many researchers argue for a master–slave type of organization between the SCN and peripheral clocks, which is a limited view in face of such complex relationship [33]. Several layers of evidence gathered over the past 20 years have created a consensus in the literature regarding such a hierarchical—top to bottom—control of the SCN over peripheral clocks. Physical disruption of the SCN leads to arrhythmic locomotor activity profiles in rodents kept in constant conditions (DD) or under a light or dark cycle (LD) [3, 34] as well as other important biological functions [35–38]. However, most of experimental data that led to the creation of this model is based on SCN lesion experiments. Such lesions not only disrupt neuronal connections of SCN but may also affect SCN neighboring connections to other parts of the brain (reviewed in [39]). In addition, evidence arisen from studies in early 2000’s, provided some findings that are difficult to fit into a top to bottom model of organization, such as the fact that timely given food affects peripheral clocks without altering the SCN [40–43]. Novel experimental tools such as tissue-specific genetic deletion indeed led to changes in the understanding of how the SCN and its surrounding structures regulate circadian rhythms. Mice with *Bmal1* deletion in SCN neurons [44, 45] or in the forebrain [46] show rhythmic locomotor activity in LD that is lost in DD [44, 45]. Gene expression data of peripheral clocks of these mice kept in LD also show a rhythmic pattern, while in DD peripheral clock gene expression rhythms are rapidly dampened [44]. Such dampening is paralleled by dampening of corticosterone secretion rhythms [44]. Bioluminescence analyses in tissues from forebrain *Bmal1* knockouts (KOs) showed that decreased amplitudes of peripheral clock oscillations upon release into DD was due to impaired cellular rhythmicity and higher phase desynchrony between single cellular clocks. Moreover, time-restricted feeding in forebrain KO mice kept in DD rescues peripheral clock oscillatory responses in the liver, but not in heart, lung, and spleen [46].

**Fig. 2:**
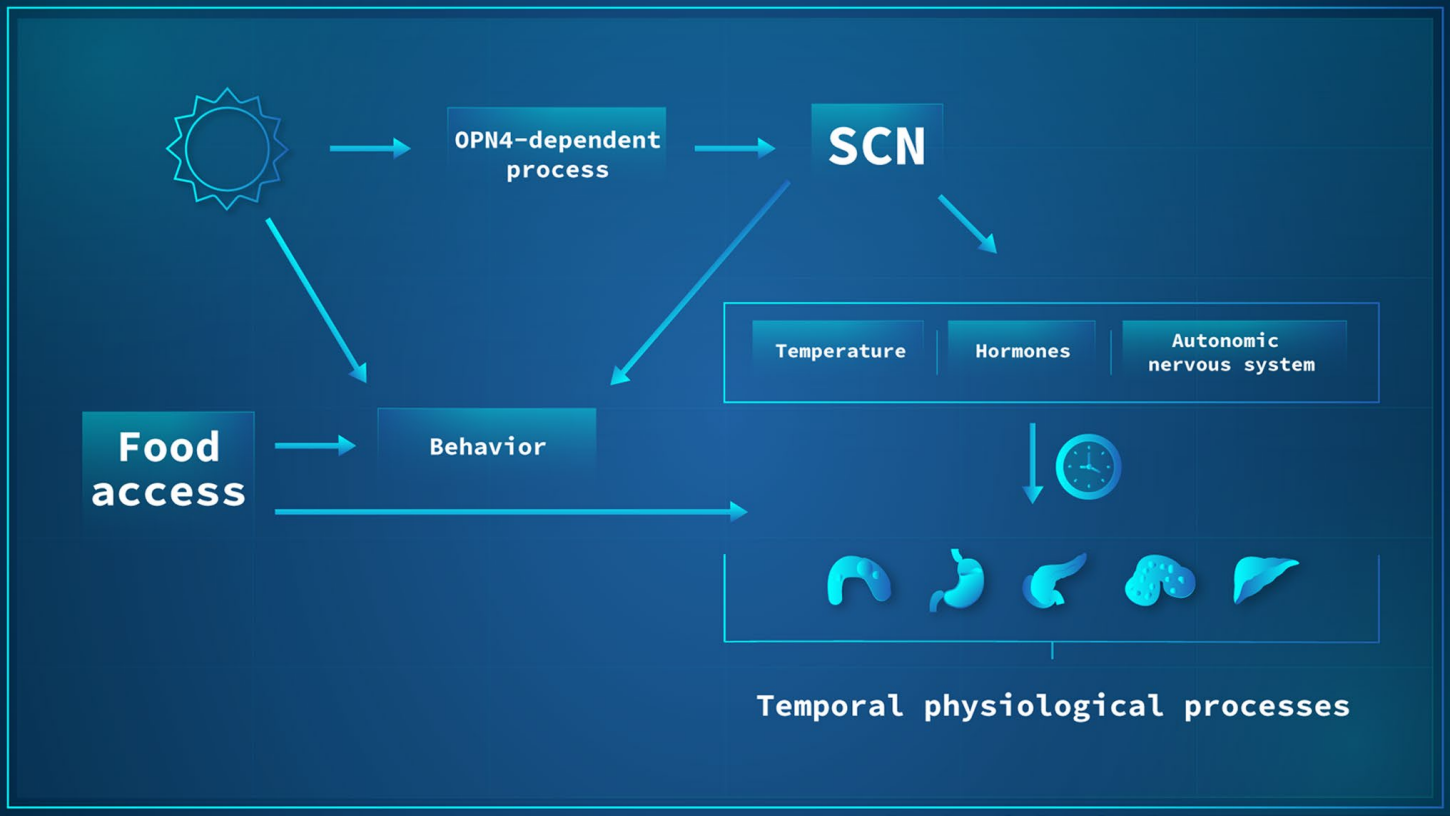
Current model of circadian network organization in mammals. The suprachiasmatic nucleus (SCN) is regulated by environmental light that is sensed by OPN4 in ganglion cells of the retina. Light information is transformed into electrical stimuli that reach the SCN. Upon SCN synchronization, the organism uses redundant temporal timing cues such as temperature, hormones, and autonomic nervous input to ensure systemic synchronization of biological processes across organs. In addition, behavior can be modulated by a direct effect of environmental light, the SCN, or by food availability which, in turn, can directly affect the molecular clocks of peripheral organs

The above-described studies demonstrate that in the absence of a functional SCN clock, peripheral clocks still entrain when mice are kept in LD. In *zeitgeber* free conditions, the SCN plays a vital coordinating role in sustaining internal rhythms. Such data argue for non-SCN regions that receive input from the retina and are able to share such information with other regions in the brain and peripheral organs, thus sustaining internal synchrony (reviewed in [39]). Thus, a new model of organization has been brought forward, the “Federated Model”. It suggests that the SCN is only required to sustain the rhythms of the organism in the absence of *zeitgebers* or under partially conflicting *zeitgeber* conditions, which resembles a hierarchical structure. This situation changes when reliable *zeitgebers* are present, when peripheral organs are able to sustain their rhythms in a SCN-independent fashion, through a tissue-specific combination of *zeitgebers*, which is expected to allow for more flexible entrainment under complex *zeitgeber* conditions than a strict hierarchical structure (Fig. 2) (reviewed in [39]).

### Chronodisruptive environment and its impact on the clock

Our current society’s living conditions are strikingly different from the early days of *Homo sapiens* [47]. With the implementation of widespread illumination in the late 1870’s, the presence of light during the evening became a reality. One may not overcast the advancement that lighting made for mankind, but it should be stressed that its putative deleterious effects may have been overlooked in the past decades. In this new environment in which light is strongly present, an important consequence takes place: reduction of the regulatory role of the light/dark cycle as a circadian *zeitgeber*. In this sense, social cues have increased their effect on human rhythms and some authors consider that we are currently living in a constant social jetlag paradigm (reviewed in [11]).

Advancements made in the food industry during the last decade have led to a state of 24-h food abundance. Moreover, modern food items are often loaded with fat and sugar. As an additional confounder, our current lifestyle is often associated with a lack of exercise as well as working in shifts [2, 11]. Therefore, the conditions in which our biological clock evolved are vastly different from the ones we face now. The biological clock is programmed to anticipate physiological events based on predictable environmental factors that include mainly light, temperature, food, and activity. However, our current society’s lifestyle poses as a challenge to this system since we have a combination of factors that negatively affect our clock. Overall, the presence of light at night, continuous availability of high-fat and -sugar food, which can be associated with a sedentary lifestyle, and the presence of night shift works, result in a condition of chronodisruption of which we currently do not fully comprehend the consequences to human’s health.

The last decade witnessed a worldwide movement to replace traditional lamps for LEDs due to their high energy efficiency [48]. However, such LEDs display a sharp blue emitting peak that is able to influence our circadian system via OPN4-detection in the eye [49] and may promote eye-related pathologies [50]. However, there are a scarce number of studies with appropriate control to evaluate the effects of LED *vs.* traditional lamps, which is acknowledged by the European Scientific Committee on Health, Environmental and Emerging Risks (SCHEER). However, SCHEER also acknowledges the effects of LEDs on the circadian system via melatonin suppression in controlled studies. Due to insufficient data in humans and appropriated controls in the studies, SCHEER reached to the conclusion of no evidence of direct adverse health effects from LED emission devices [51]. Nevertheless, SCHEER also recognizes that LED technology is still growing and a closer attention to its long-term effects is required [51].

The best example of chronodisruption in humans are shift workers. An increasing number of epidemiological studies show an association of shift work with the development of several diseases (reviewed in [52, 53]). Remarkably, very recently the International Agency for Research on Cancer (IARC) kept the same recommendation made in 2007 that shift work in humans is “probably carcinogenic to humans”, evidence grade 2A, i.e., with sufficient data in experimental models but limited in humans, mainly due to a higher variability between the studies [54, 55]. Moreover, studies that combine satellite images with epidemiological data suggest a correlation between light at night (LAN) exposure with increased incidences of breast cancer—but not with colorectal, larynx, liver, and lung cancers [56]—as well as overweight and obesity [57]. Nevertheless, conclusive data about LAN effects are still pending. Despite the lack of hard evidence for the deleterious effect of shift work and LAN in humans, it is clear that the conditions of our society are completely different from the ones in which biological clocks evolved [1, 47].

Based on such knowledge, it is tempting to link the increasing incidence of several diseases such as metabolic, cardiovascular and neurological disorders, and cancer in humans to a possible disruption of the circadian clock (reviewed in [1, 2, 10, 47, 58, 59]). Ultimately, the consequences of chronodisruption to human health are still being comprehended—it is an ongoing field of investigation. However, the more we understand the long-term consequences, the better policy makers can act, which could potentially prevent a substantial loss of life and provide a better life quality for large parts of the population.

### Communication between central and peripheral clocks: a one-way street?

#### Overview of SCN-driven inputs to peripheral clocks

The pathways used by the SCN to relay temporal information to the organism comprise of complex and partly redundant signals which can be divided into four categories: sympathetic and parasympathetic nervous stimuli, hormonal outputs, feeding-fasting rhythms, and circadian oscillations of core temperature rhythms (Fig. 2). SCN efferent projections have been shown to terminate in several brain regions such as sub-paraventricular zone, the preoptic area, the nucleus of the stria terminalis, the lateral septum, the dorsomedial hypothalamus, the arcuate nucleus (ARC), and the paraventricular nucleus (PVN reviewed in [6, 8, 31]). In addition, the SCN receives input from hypothalamic and extra-hypothalamic regions, which also are important for regulating SCN physiology [6, 60].

The first pathway is autonomic output from the SCN that leads to rhythmic clock gene expression [61]. However, autonomic denervation is not required for rhythmic gene expression in the liver [62], thus arguing for additional routes of synchronization. Interestingly, SCN-driven sympathetic outputs have been shown to decline with aging [63].

A second pathway is comprised of hormonal outputs mainly comprised of the pituitary–adrenocortical axis (HPA), specifically via glucocorticoids. Corticoliberin (CRH) secretion by the PVN exerts a control over the rhythmic release of adrenocorticotropic hormone (ACTH) in the pituitary. Through downstream signaling by the pituitary, glucocorticoids are rhythmically released by the adrenal cortex peaking at the beginning of the active phase [35, 64, 65]. Glucocorticoid action takes place through mineral (MR) and glucocorticoid receptors (GR), whose expression is widely found in peripheral tissues [66]. It is worth mentioning that the promoter region of the *Per* genes contains glucocorticoid response elements (*GRE*s) whose activation leads to gene transcription and provides a molecular basis for glucocorticoid action in the circadian machinery [67]. Another classical synchronizing hormone is melatonin, which (known as the hormone of darkness) is secreted exclusively during the night in both nocturnal and diurnal species. Upon light exposure, the SCN sends inhibitory information to the PVN region, leading to suppression of melatonin synthesis. In the dark, a glutamatergic activation of the PVN leads to norepinephrine release in the pineal gland and subsequent melatonin synthesis (Reviewed in [68]). Melatonin conveys temporal information to peripheral organs through its interaction with its G-protein coupled receptors (MTNR1A/B or MT1/2) found in peripheral organs (reviewed in [68, 69]). Melatonin suppression by light at night (LAN) as result of modern lifestyles [1] is associated with several diseases, probably through its disruptive effect on the circadian clock network [70, 71].

A third pathway involves feeding-related activities. Temporal food restriction is an important *zeitgeber* for metabolic tissues, being able to synchronize local clocks while exerting few effects on the SCN [41, 42, 72]. Of note, though, combining timed feeding with hypocaloric diet regimes can affect SCN activity and SCN-driven outputs such as locomotor activity, melatonin synthesis [73, 74]. In rodents, very restrictive feeding regimes also led to alterations in SCN clock gene expression, though SCN outputs were not evaluated in these studies [75, 76].

The last pathway used by the SCN is internal temperature rhythms. Thermoregulatory coordination processes are centralized in the hypothalamic preoptic area in which the median preoptic nucleus is a key player [77, 78]. Core body temperature increases and decreases before the onset of the active and resting phases, respectively. Such anticipatory regulation is controlled by the SCN [79]. Along the day, core temperature is known to be rhythmic oscillating from 36.5 to 38.5 °C with a mean of 37 °C in humans and rodents [80–82]. Mounting evidence from in vitro studies has demonstrated that tissues and cells of peripheral clocks are able to respond to cold or warm temperature when presented as a short pulse or cycles. Thus, diurnal variations in body temperature are assumed to play an important role in peripheral clock synchronization [83–87]. The receptors responsible for detecting thermal energy in peripheral tissues are still elusive. However, the large family of transient receptor potential (TRP) channels [88–90] and, more recently opsins, whose thermo-detecting capabilities have been demonstrated in mammals, are interesting candidates that may detect temperature fluctuations to reset molecular clocks [83, 91, 92].

### Overview of peripheral inputs affecting the SCN

The SCN is considered largely resistant to potential synchronizing factors other than light. For instance, glucocorticoids display no or only indirect effects on the SCN due to an absence of GR receptors in this area [93, 94]. The same holds true for temperature changes since the SCN is able to compensate these effects [86, 95]. The insensitivity of the SCN to synchronizing factors is important for its role as a central oscillator, though this does not mean that it cannot integrate feedback factors to adapt systems rhythms.

One of such factors, leptin, the satiety hormone, displays peak blood levels at night in nocturnal rodents, which are controlled by SCN-driven sympathetic innervation of adipose tissue [96]. In vivo leptin injections at the beginning and the end of the diurnal activity phase do not affect locomotor activity of mice kept in either LD or DD conditions. However, leptin augments the phase-shifting effects of light pulses during subjective night specifically in females [97]. Vice versa, in leptin deficient *ob/ob* mice, leptin administration normalizes photic responses at the behavioral and molecular level [98]. Another important metabolic hormone, ghrelin that is synthetized by the oxyntic gland cells of the stomach participates in the anticipation of feeding time by activating its receptors in the ARC [99, 100]. In addition, ghrelin positive neurons in the hypothalamus receive direct input from the SCN [101]. In vivo experiments reveal that ghrelin, when given to animals kept under an *ad*-*libitum* diet, does not affect locomotor activity. However, upon food deprivation ghrelin analogs phase advance locomotor activity in mice in DD [102]. Of note, the SCN is resistant against insulin-induced clock resetting [103].

Hypocaloric restricted feeding regimes display a strong effect on the SCN [73, 74]. In addition, long-term high-fat diet (HFD) conditions influence the SCN of male mice [104, 105], but not of females [106, 107]. One week-long HFD does not affect SCN rhythms but disrupts liver clock regulation [108]. Remarkably, the starvation signal, fibroblast growth factor 21 (FGF21), a liver-born molecule, leads to decreased systemic insulin levels and locomotor activity, and increased corticosterone, which represents a classical starvation response. Such responses were dependent on the presence of β-klotho in the SCN and in the dorsal vagal complex of the hindbrain [109]. Moreover, β-hydroxybutyrate (β-OHB), a liver-borne molecule, has been implicated as a player in food-anticipatory activity (FAA) response via a putative interaction with brain regions, which are still elusive [110].

Melatonin, whose receptors are present in the SCN, has important effects on neuronal firing and clock gene expression in the central pacemaker [111–113]. Additionally, increased blood pressure was shown to affect neuronal activity in the nucleus tractus solitarius (NTS), but also in the SCN. Interestingly, the SCN receives direct projections from the NTS and plays an important integrative and regulatory role in blood pressure circadian control [114]. Physical exercise does not affect the SCN, but it resets many peripheral clocks [115]. More recently, the presence of a non-metastatic melanoma tumor in mice was shown to reduce *Bmal1* and increase *cFos* gene expression in the SCN compared to control animals, suggesting that molecules released from the tumor may affect the SCN as well peripheral tissue clocks [116].

#### Relative autonomy of peripheral tissue clocks

Global knockout mice have allowed a substantial increase in our knowledge regarding gene function. However, with regard to the circadian clock network, this strategy is limited since gene deletion is global and do not allow dissecting the effects of specific tissue clocks on physiological functions. Such limitation was circumvented by tissue-specific gene knockout strategies such as the Cre/loxP system, which can also be conditional, i.e., gene deletion may be induced at a specific timepoint of development (Reviewed in [117]). Regarding the molecular clock, only the *Bmal1* deletion results in loss of rhythms as other clock genes are functionally compensated by their paralogs which makes dual knockouts necessary, which is the case for *Per* and *Cry* genes (reviewed by [118]). So far, an increasing number of studies using global and tissue-specific knockouts has allowed our current understanding regarding the functional organization of the clock network in both physiological and pathological scenarios.

Early organ transplantation experiments between clock gene mutant and wild-type animals have been key to understand circadian organization at the network level [3, 65]. More recent advancements in molecular biology allowed studying the molecular clock in a single organ while the others remain without a functional clock machinery. In these landmark studies, using a conditional gene trap strategy, in mice lacking *Bmal1* in all tissues, *Bmal1* expression and, thus, clock function is restored upon Cre expression in either liver or the epidermis of the skin [119, 120]. In the liver clock rescue mice, glycogen synthesis and nicotinamide adenine dinucleotide (NAD +) salvage production are rhythmic, but only when mice are kept in LD. Thus, the liver clock is able to sustain its autonomy despite the absence of functional clocks in the rest of the organism—including the SCN. Rhythmic hepatic lipid and xenobiotic metabolites are not restored, though. In line with this, compared to wild type mice, reconstitution of the molecular clock of the liver rescues rhythmicity in approximate 10% of gene transcripts and 20% of metabolites. These data show that most rhythmic processes of the liver are not autonomous and dependent on systemic time cues that only exist in an intact circadian organism. Along this line, liver clock restoration does not revert the reduced life span of global *Bmal1* KO mice [119]. In an accompanying study, clock gene restoration of the epidermis yielded similar findings to the ones described above. Upon epidermal clock rescue, approximately 15% of transcriptional rhythmicity is restored. Again, all rhythms are completely lost in DD [120]. Overall, local tissue clocks in the epidermis controls basic tissue functioning, i.e., epidermal turnover. Epidermal clock restoration does not rescue the reduced life span of *Bmal1* KO mice, but it inhibits premature skin aging [120].

This new strategy has opened a new investigative field, which will allow the precise contribution of each organ system to the overall circadian organization. Importantly, both studies do not establish whether the arrhythmicity found in the clock rescued liver or skin in DD is due to complete loss of rhythms at single cell levels or due to a loss of coupling (*i.e.,* circadian communication between cells), which ultimately leads to an apparent loss of rhythms. At the same time, it emphasizes the key role of light in clock resetting—even at the peripheral tissue level. One may argue that mouse skin cells, which are known to be photosensitive [121–125] could be responsible for synchronizing the local clock in vivo. Indeed, such concept has been proven as neuropsin (OPN5) participates in light-induced clock gene synchronization of the skin [124]. This does obviously not apply for liver, though. Studies from our lab had previously shown that light synchronizes peripheral clocks in the absence of a functional SCN, thus suggesting that other brain regions that receive light input via retina may ultimately sustain circadian rhythms of the organism [44, 45]. In summary, these findings argue for a less SCN-centric organizational view of the circadian clock system, which agrees with the federated model of organization [39].

Very recent experiments provide evidence that liver or fibroblasts from *Bmal1* KO mice, when cultivated ex vivo and synchronized with dexamethasone, indeed display robust rhythms in gene and protein expression for days. The authors also ruled out a putative compensatory function of *Bmal2* (*Arntl2*). Interestingly, *Bmal1* KO cells displayed almost no overlapping rhythmic transcripts and proteins compared to wild-type cells. Indeed, in the absence of *Bmal1* a new set of transcription feedback loop is recruited controlling novel and non-canonical molecular rhythms [126].

Taken altogether, systemic cues are important to drive circadian rhythmicity of liver, skin, and likely all other organs. However, local clocks can sustain rhythmicity of some biological processes even in the absence of a functional circadian network. It will be of great importance to identify the mediators of circadian network timekeeping as they make great targets for chronotherapies. In addition, a novel non-canonical clock was reported in the absence of *Bmal1*. Such processes may be of importance in diseases in which circadian disruption alleviates physiological control by the clock machinery.

#### Putative mediators of inter-tissue communication

As described previously**,** we have uncovered how SCN signals to periphery as well as how periphery may affect SCN functioning. In this section, our aim is to provide an overview of putative signals released by peripheral organs and how they may affect clock gene functioning of peripheral tissues.

**Fig. 3:**
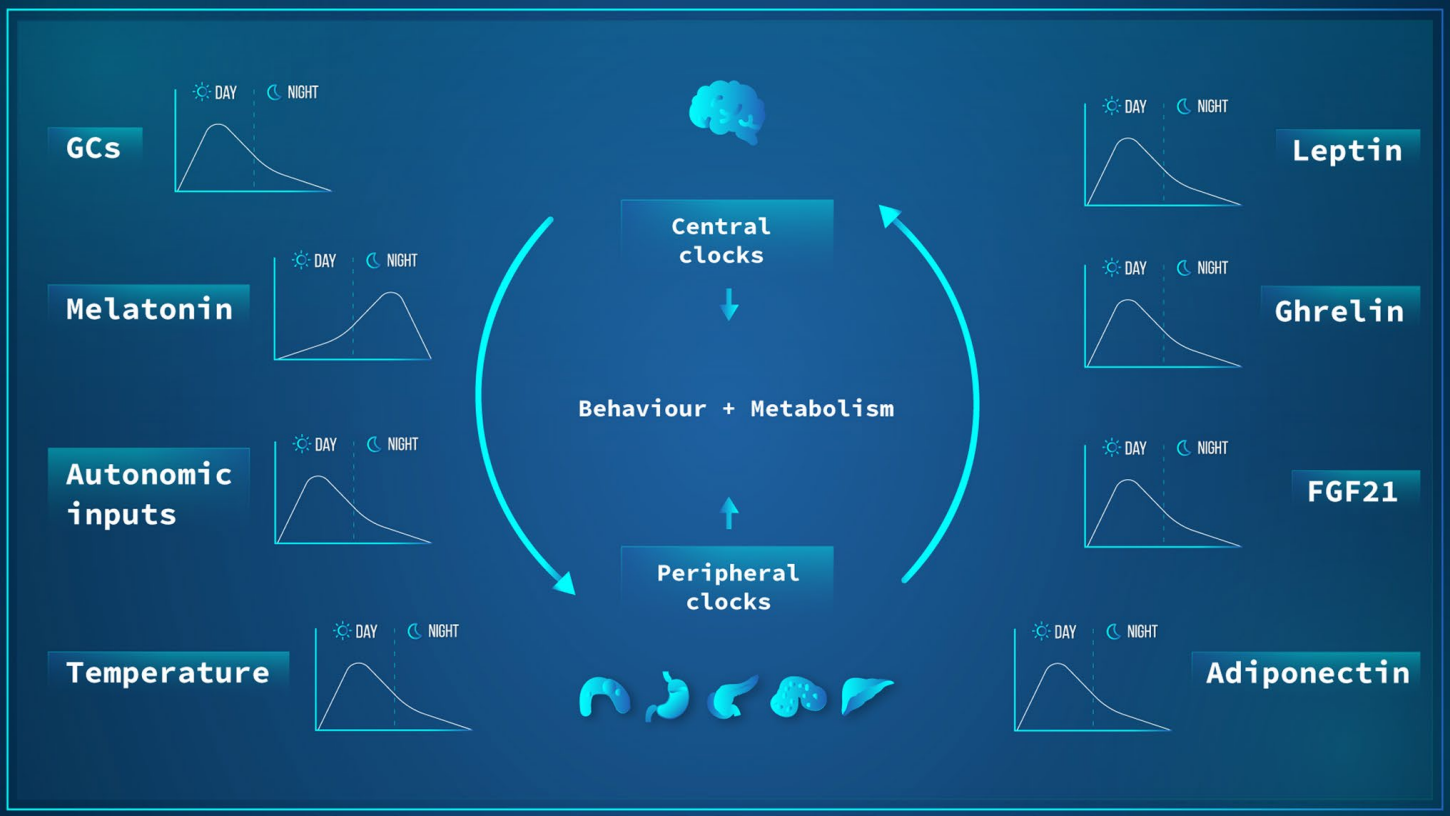
Bidirectional communication between central and peripheral tissue clocks. Temporal cues controlled by the central pacemaker (SCN) and other CNS clocks such as glucocorticoid and melatonin secretion, autonomic inputs, and body temperature are known to affect the molecular clocks in peripheral organs. At the same time, peripheral clocks, through molecules like leptin, ghrelin, FGF21, and adiponectin can feed back on the SCN and other brain region clocks. The overall result of this entangled communication network is an integrated rhythmic control of behavior and metabolic outputs. In this figure, it is didactically represented the phase (day or night) in which each factor has its highest value in humans

Based on a decentralized circadian organizational view, one may expect the presence of redundant signals that arise from: (1) the SCN to periphery; (2) periphery to SCN, (3) and between peripheral organs. Collectively, these pathways sustain the circadian network organization (Fig. 3). Twenty-four hours long metabolomic data of SCN, medial prefrontal cortex, liver, muscle, sperm, WAT, BAT, and serum demonstrated an extensive communication network between organs to sustain coherence of the temporal metabolic pathways. Remarkably, such coordination and inter-tissue metabolite correlation and coordination is lost and rewired by high-fat diet. Interestingly, while some tissues show partial gain or loss of metabolites rhythms, the serum metabolites are the most affected, i.e., being the most affected (loss of correlation) under HFD [127].

The identification of putative players responsible for regulating the molecular clock of peripheral organs is still largely elusive. Indeed, the role of several gastrointestinal peptides such as cholecystokinin (CCK), gastrin, ghrelin, glucose-dependent insulinotropic polypeptide (GIP), motilin, neurotensin, neuropeptide Y family, secretin, and VIP in regulating circadian rhythms have been suggested but experimental evidence is still lacking (reviewed in [128]). On the other hand, oxyntomodulin (OXM), a glucagon-like hormone, administration to liver explants was shown to affect the molecular clock and carbohydrate-related CCGs as well as when given in vivo. Interestingly, blockade of OXM suppresses food-induced molecular clock alterations in the liver—but not in the SCN—thus placing OXM as a direct link between food-related effects in hepatic circadian clock [129]. Ghrelin administration to steatotic liver was shown to restore circadian rhythmicity in in vitro and in vivo in an mTOR-dependent fashion [130]; however, the role of ghrelin in a physiological setting is still unknown.

Insulin and glucose are classical players in modulating the molecular clock of metabolic and non-metabolic organs. For instance, insulin and insulin-like growth factor-1 (IGF-1) affect both the phase and amplitude of clock genes in in vitro, ex vivo, and in vivo through increased PER transcripts of liver and kidney. Such response was not dependent on the classical clock genes and the SCN was insensitive to the insulin-dependent effects [103]. It has been suggested that insulin is an important player in food-induced clock gene alteration in the liver as streptozotocin-treated animals, i.e., without insulin production, are not affected by food regimes while exogenous insulin rescued the effects of food in the hepatic clock [103].

Indeed, insulin has been shown to signal through phosphatidylinositol 3-kinase (PI3K) and Forkhead box class O3 (FOXO3) signaling to sustain liver rhythms in a *Clock*-dependent fashion [131]. Insulin has also been shown to phosphorylate BMAL1 in a protein kinase B (AKT)-dependent process, which results in BMAL1 dissociation from the DNA, and consequently, a reduction in its transcriptional activity [132]. Moreover, cAMP Responsive Element Binding Protein 1 (CREB)/ CREB regulated transcription coactivator 2 (CRTC2) role has been suggested in liver clock synchronization during fast and feeding. During fast, glucagon activates CREB/CRTC2 that ultimately induces *Bmal1* transcription while insulin, on the other hand, suppresses *Bmal1* expression by inhibiting CREB/CRTC2 activity [133]. Interestingly, CRTC2 has been shown to regulate hepatic gluconeogenesis in a cooperative fashion with glucocorticoid hormones and its receptor and glucagon via CREB-dependent pathway [134]. Whether CRTC2-dependent interaction with glucocorticoid, whose role as a synchronizer of the hepatic clock is established [135], is still elusive.

Importantly, mTOR signaling has been suggested as a vital pathway through which peripheral clocks use to adjust their local circadian rhythms. In both central and peripheral clocks, mTOR activation results in shorter oscillatory profile, i.e., it speeds up the clock while mTOR inhibition results in the opposite effect [136]. Therefore, mTOR pathway seems to be a broad signal hub for adjusting the molecular clocks; however, the identity of players that modulate this pathway is largely unknown. Nevertheless, such widespread pathway may play useful in restoring circadian functioning in pathological conditions.

Taken altogether, the identification of peripheral clock regulators that are not directly driven by the SCN, but rather by peripheral clock themselves is ongoing field of investigation, and there are many gaps of knowledge. Indeed, one may suggest a complex and redundant communication network through which peripheral clocks communicate with each as elegantly shown by Dyar and colleagues (2018) [127]. Comprehending the role of each hormone, peptide, and/or metabolite will prove an exhausting task; however, it must be emphasized that the discovery of such players and pathways would be crucial for the development of novel pharmacological targets for metabolic diseases.

### Clock-metabolism crosstalk

#### Cellular processes

The importance of the biological clock as a temporal regulator and coordinator of complex biological processes of tissues and systems can be appreciated when key biological processes of a particular organ are comprehended. Extensively revising each biological process is beyond the scope of this review. However, in the next lines, we will highlight how the molecular clock is of importance to ensure metabolic homeostasis by controlling some important intracellular processes as well as biological processes of metabolic organs.

One of the most important clock-regulated processes is the information flow from DNA to proteins. In recent years, a close association between clock proteins and histone modifications has been unraveled contributing to the regulation of circadian rhythms at the molecular level. Specific cyclic chromatin transitions happen along 24 h and are associated with changes in transcriptional activity [23, 137–139]. Among different types of histone modification, acetylation is the most studied with regard to circadian rhythms [23].

As for the mRNA level, several lines of evidence show that most transcripts (nascent and processed RNAs) are not transcribed in a circadian fashion, thus ruling out a concept that most gene rhythms would be driven by *E-box* mediated transcription. Indeed, abundance of only approximate 25% of cycling mRNA transcripts are controlled by *de-novo* transcription, thus suggesting that post-transcriptional regulatory mechanisms are critical to generate expression rhythms for the remaining 75% of mRNAs [138, 140]. On the other hand, recent mathematical modeling has suggested that a lesser faction (~ 35%) of rhythmic mRNAs are regulated at the post-transcriptional level [130]. mRNA processing such as capping, splicing, polyadenylation, and cytoplasm exportation have been shown to be clock regulated, thus contributing to overall circadian organization (reviewed in [141]). Another important regulatory step is miRNA-mediated mRNA degradation. In this sense, a liver-specific *Dicer* knockout demonstrated that in the absence of most miRNAs, the hepatic clock machinery is still functional and shows modest alterations in gene expression in terms of phase and period. Such findings demonstrate that miRNAs may not be major driving forces of mRNA rhythms, but rather miRNAs are a fine-tuning tools of rhythmic transcriptomes [142], which is further supported by other experimental studies [143, 144].

Many studies have highlighted an important lack of concordance between rhythmic mRNA and protein levels, thus suggesting that many rhythmic proteins do not undergo an oscillation at the transcript level [130, 145, 146]. This may be explained by oscillatory rhythms in ribosome biogenesis and translation initiation complex formation [147–149] which may overcome the lack of oscillation at the mRNA level. Additionally, recent studies have revealed further post-translational modifications and their regulatory role in circadian timekeeping. Phosphoproteome analyses from liver revealed that 25% of phosphorylation sites and 40% of phosphoproteins are regulated in a clock-dependent manner [145]. In the murine hippocampus, approximately 2% of proteins and 5% of phosphorylation sites exhibit a circadian profile; however, in this study, no clock proteins were detected [150]. In mouse liver, 8% of proteins are rhythmic and are associated with immune responses, cell cycle regulation, lipid and glucose metabolism. Interestingly, almost 3,500 ubiquitination sites were found on 1144 proteins, which were enriched during daytime compared to night [151]. For a detailed view of post-translational processes involved circadian clock function, the reader is referred to a recent review [152].

**Fig. 4:**
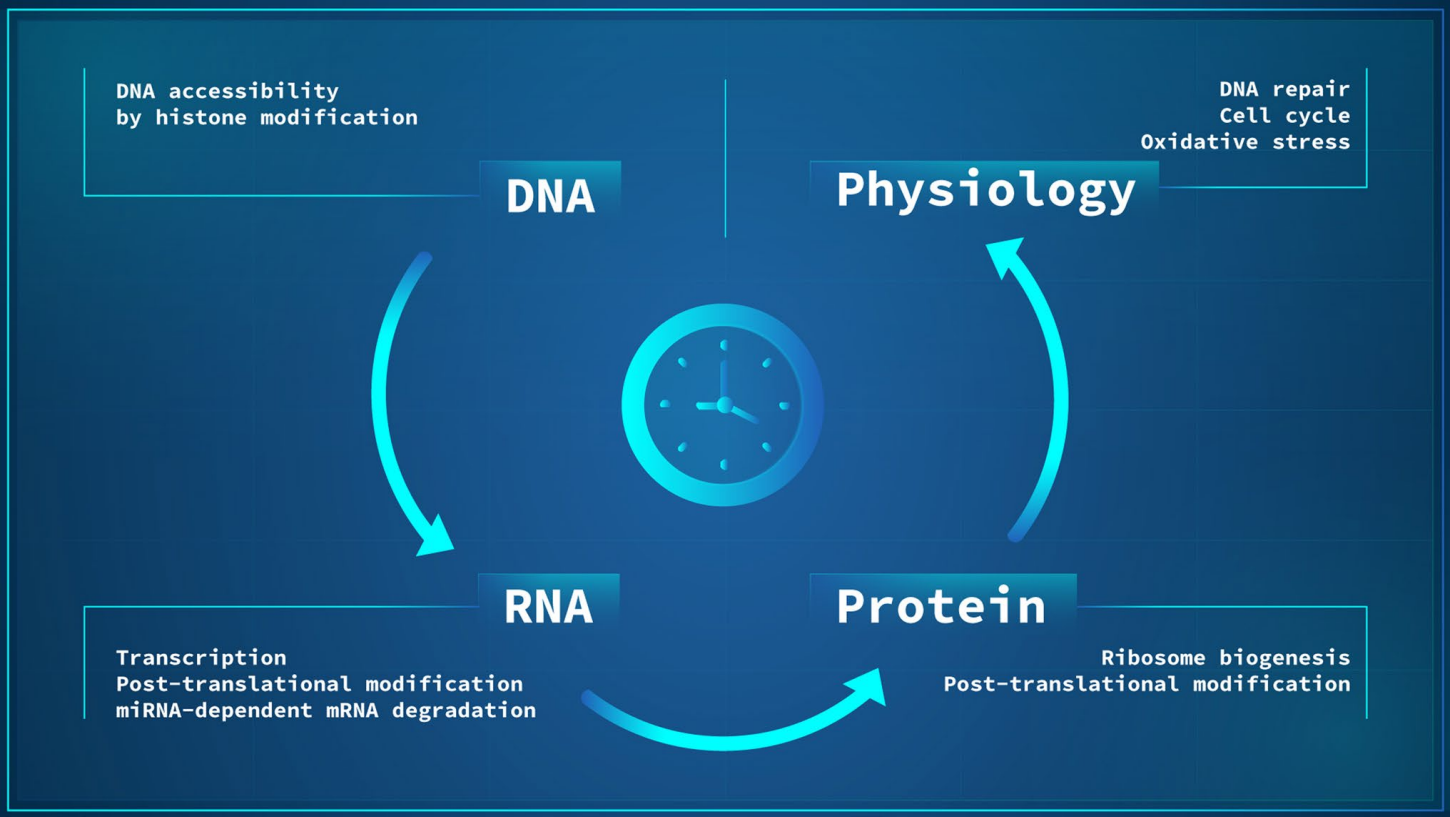
The classic cellular information flow from DNA to RNA and to protein is subject to circadian control at all levels. This rhythmic biological information flow contributes to the circadian control of cellular physiology and downstream biological processes. Among these, we highlight DNA repair, oxidative stress, and cell cycle regulation

Taken altogether, recent evidence highlights the various layers of circadian control, starting from DNA accessibility to transcription factors, a histone dependent process, which is followed by RNA transcription. Upon mRNA synthesis, several regulatory (post-transcriptional) steps need to be met, and most of them are subject to circadian control. Finally, information is translated from mRNA to protein, which also undergo several post-translational modifications that directly affects biological function. The increasing view that *de-novo* transcription accounts for just a fraction of rhythmic gene expression, a long-standing concept in the field, associated with the lack of overlap between rhythmic genes and proteins, place post-transcriptional and translational modifications as important regulatory steps of the circadian clock (Fig. 4), whose role and functions are still being understood.

The **cell cycle** consists of three main processes that include a DNA replication step (S phase) that is followed by a stage of mitosis (M Phase), and in between growth stages (G1 and G2) in which cells prepare themselves for the next division. In G1, cells prepare for DNA replication by increasing protein synthesis and cell growth. In G2, growth-related processes also take place in addition to the DNA damage check point, a fundamental step to avoid wrong, potentially deleterious, information to be passed on to daughter cells. Sequentially speaking, dividing (non-quiescent) cells start increasing their size (G1), which is followed by DNA replication (S Phase) and another growth phase (G2) that is followed by mitosis (M Phase). Quiescent cells (G0) may enter the cell cycle depending on a series of environmental factors. Senescent cells, on the other hand, are terminally differentiated and cannot re-enter the cell cycle. The progression through each stage is strongly controlled and dependent on a series of activations of cyclin-dependent kinases (CDKs) that form specific complexes with cyclins (CCNs) for each cell cycle transition [153–155]. Elegant in vitro studies have demonstrated that in free-running conditions there is a robust coupling between cell cycle and circadian clock. Interestingly, in active dividing cells a shortening of clock period happens when compared to non-dividing cells, thus suggesting an influence of the cell cycle on the molecular clock [156, 157]. On the other hand, in synchronized cells, two major events take place: a fraction of cells remains phase locked, i.e., one cell cycle per one clock cycle while another sub population displays three cell cycles for each two clock cycles [157]. Taken together, these studies suggest that in absence of synchronizers (free-running conditions), the influence of the cell cycle on the molecular clock is dominant. On the other hand, in synchronized conditions the circadian clock displays a dominant role over the cell cycle. Some researchers have suggested that the molecular clock is able to “gate”, i.e., allow cell cycle progression [158], a concepted that has been challenged [156, 157, 159]. The molecular clock exerts its effects on the cell cycle either by controlling transcription of key regulatory genes or by protein–protein interaction (reviewed in [160]). In this line, it is not surprising to realize that disruption of the circadian clock is in fact correlated to a loss of cell cycle progression control, often seen in cancer [161] [162–164].

In an elegant and complex process, DNA repair can revert DNA damage caused by a myriad of genotoxic agents. There are several types of DNA repair in mammals such as direct repair (by alkyl transferases), base excision repair (BER, by glycosylases and AP endonucleases), double-strand break/crosslink repair, and nucleotide excision repair (NER) [164]. Available evidence suggests that NER is strongly controlled by the clock. NER-dependent DNA repair is carried out by six factors: replication protein A (RPA), Xeroderma Pigmentosum Complementation Group A, (XPA), Xeroderma Pigmentosum Complementation Group C (XPC) complexed with RAD23 Homolog B (HR23), Transcription factor II Human (TFIIH), Xeroderma Pigmentosum Complementation Group G (XPG), and Xeroderma Pigmentosum Complementation Group F (XPF). For a detailed view of this process the reader is referred to in-depth reviews [164–166]. In pioneering studies led by Aziz Sancar’s group, NER mediated activity was shown to be rhythmic in brain [167] and liver, but not in testis [168] and skin [169]. In these tissues, XPA was the only NER-related protein to show an oscillatory profile, in antiphase to CRY1 [167–169]. In the absence of CRY1/2, such rhythmic profile of XPA was abolished in liver and skin [168, 169]. In terms of functional activity, excision repair of cisplatin adducts in the liver and UVB-induced DNA damage are highest in the afternoon (4–5 pm) and lowest in the morning (4–5 am), thus highlining important findings for chronomodulated therapies [168, 169]. Moreover, UVB-induced erythema is maximal in the morning *vs.* exposure in the afternoon in mice, which is associated with reduced DNA repair in the early morning compared to the afternoon [170]. Indeed, such concept has been translated recently to humans as UVB-induced erythema was shown to be milder in the morning compared to evening exposures [171]. Some studies have suggested that BER-dependent activity may also be influenced by the molecular clock. BER-mediated DNA repair activity has also been suggested to display a rhythmic profile in humans as the expression of 8-oxoguanine DNA glycosylase (*OGG1*) is rhythmic. Oxidative DNA repair in lymphocytes is higher in the afternoon compared to the morning. In vitro experiments show that *Bmal1* knockdown leads to loss of rhythmic expression of *OGG1* [172]. In addition, in hepatocytes, BER activity, via N-methylpurine DNA glycosylase (MPG) protein expression, is rhythmic and depends on CLOCK [173].

Controlled generation of reactive oxygen and nitrogen species (ROS/RNS) in a cell is an important step for cellular signaling processes since these molecules may act as secondary messengers. Disruption of this rigid control may result in increased ROS levels, which may give rise to oxidative stress, damage macromolecules, and ultimately promote apoptosis or necrosis (reviewed in [174]). Cellular ROS is predominantly generated as superoxides because of mitochondrial oxidative phosphorylation. Detoxification of ROS takes places via a complex set of enzymes such as superoxide dismutases (SODs), catalase, glutathione peroxidase (GPx), and free radical acceptor molecules like peroxiredoxins (PRDXs), glutathione (GSH), or thioredoxin (TRX) (reviewed in [174, 175]). Without a proper control of ROS/RNS generation and detoxification, the consequences for cellular fate can be disastrous. Indeed, chronic oxidative stress has been implicated in the development of several diseases such cardiovascular, endocrine, aging, neurodegenerative, cancer, and many others [174, 176, 177]. Since ROS is linked to cellular metabolic state, which itself is known to undergo an oscillatory rhythm, the generation of ROS and the expression of detoxification enzymes have been shown to oscillate along the day in mammals [178–183]. Indeed, the presence of *E-box* elements in promoter regions of several detoxification enzymes has been shown in humans and mice, thus suggesting a possible route of ROS control via transcriptional activity by the molecular clock [184]. Interestingly, circadian control of oxidative defenses was shown to take place in absence of transcriptional activity in red blood cells [185]. For a more in-depth information, the reader is refereed to excellent reviews on this topic [186, 187].

#### Metabolic tissue circadian functions

**Fig. 5:**
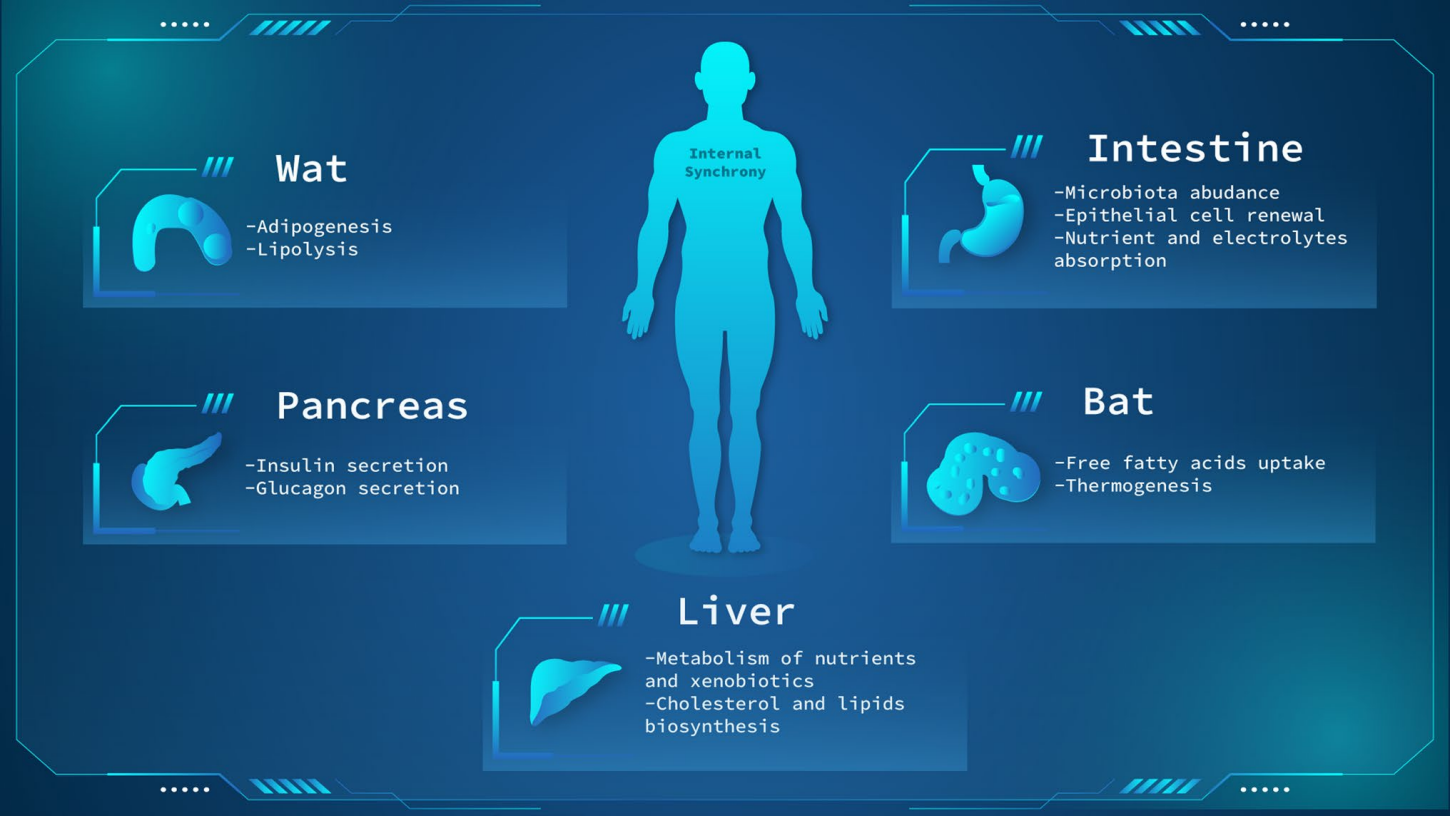
Overview of the main clock regulated biological processes of energy metabolic tissues. Each organ is represented in the figure with major clock-dependent biological functions

In the last section, we will provide an overview of clock regulated biological processes in metabolic organs (Fig. 5) and some recent advancements made in the field.

**White adipose tissue (WAT)** is the most abundant type of adipose tissue in mammals and has traditionally been associated with its energy storage capacity in form of triacylglycerol (TAGs). In this process, TAGs are released from WAT via lipolysis to generate free fatty acids (FFAs) and glycerol, which are taken up by other tissues to generate energy [188]. The WAT molecular clock has been shown to control the above-mentioned process of lipolysis [189, 190]. In addition to its classical energy storage capacity, it has become increasingly recognized that WAT is an active endocrine organ able to synthesize several biologically active molecules, which may regulate homeostasis [191]. Several studies have demonstrated rhythmic expression of clock genes in WAT of different origins in mice [189, 192–194] and humans [195–197]. Moreover, microarray and transcriptomic analyzes have demonstrated that hundreds of genes show an oscillatory profile in mice [24, 193, 198] and humans [196, 199, 200], many of which are involved in key physiological processes such as lipolysis, adipogenesis, and energy conversion. The role of the molecular clock in regulating WAT physiology can be clearly seen in clock gene knockout mice. Global deletion of *Bmal1* or *Rev-Erbα* inhibits [201, 202] while *Per2* or RORα/γ knockouts favor adipogenesis [193, 203]. Along this line, peroxisome proliferator-activated receptor gamma (PPARγ), a classical clock-controlled factor, plays a pivotal role in adipocyte differentiation [204, 205]. Interestingly, rhythmic expression of core clock genes and *Ppar*γ is severely dampened in experimental models of HFD [105]. However, female mice are protected from the deleterious effects of HFD [106, 107] which seems to be associated with smaller effects on the molecular clock machinery [206]. In the clinical setting, the therapeutic value of PPAR agonists is clear as fibrates (targeting PPARα) are known to reduce plasma lipids while thiazolidinediones (PPARγ) improve insulin sensitization in diabetic patients [207]. Moreover, clinical investigation of dual or pan-PPAR activators for treating metabolic-related disorders are currently being evaluated [207]. Global clock gene knockout is often associated with altered WAT functioning and body weight, which is gene-dependent (reviewed in [208]). As data from global knockout mice suffer from several limitations, the physiological role of each clock gene in a particular organ might be over- or underestimated. In recent years, adipocyte-targeted clock gene deletion has rendered interesting results. Adipocyte protein 2 (*aP2*) gene-Cre driven *Bmal1* removal leads to increased food consumption and body weight—regardless of diet type. In addition, mice with targeted *Bmal1* deletion in WAT show altered polyunsaturated fatty acid serum levels, which affects central mechanisms of appetite regulation in the hypothalamus. Indeed, non-obese patients display rhythmic serum levels of leptin and adiponectin [209], which are dampened in obese subjects [210]. A specific WAT *Bmal1* deletion under adiponectin (*Adipoq*) Cre control has a mild phenotype and regular body weight on standard diet, but displays a higher probability to become obese under HFD [190]. WAT of mice with targeted SCN clock gene disruption show arrhythmic expression of genes related to lipid and carbohydrate metabolism. However, in this model genes involved in immune response gain rhythmicity in the absence of an SCN clock [198]. In humans, single nucleotide clock gene polymorphisms (SNPs) have been associated with development of metabolic disorders (reviewed in [211]). Moreover, loss of clock gene rhythms in type-2 diabetic patients has been shown when compared to healthy individuals. Interestingly, in diabetic patients, loss of rhythms in lipolysis was also found [200].

**Brown adipose tissue (BAT)** is a specialized thermogenic organ that can convert chemical energy into heat and recently. BAT has recently moved into the focus of translational research due to its putative role in fighting obesity and diabetes [212]. BAT thermogenic activity is triggered by cold stimuli in an adrenergic-dependent pathway, which results in lipolysis. FFAs are transported into mitochondria in which they undergo β-oxidation. In BAT, this process does not lead to energy generation, but rather results in non-shivering heat generation. This is possible due to uncoupling protein 1 (UCP-1), which creates proton leaks in the respiratory chain, thus generating heat instead of energy [213]. Interestingly, circadian transcriptome analyzes have revealed that approximately 10% of protein-coding genes [24] as well as approximately 40% of nuclear receptors in BAT oscillate within a day [214]. Glucose levels, FFAs uptake, and clearance of serum lipids in BAT is rhythmic [215, 216]. Interestingly, mice exposed to a cold stimulus at 5 a.m. respond better compared to controls exposed at 5 p.m. due to reduced *Rev-erbα* expression in the morning. Remarkably, loss of *Rev-erbα* improves cold tolerance. *Rev-erbα* knockout mice show increased UCP-1 levels and loss of rhythmic body temperature and BAT activity [217]. In *Rorα* knockout animals increased UCP-1 levels and browning of WAT is found in addition to a higher ex vivo respiration in both BAT and WAT compared to control [218]. On the other hand, *Per2* knockout mice are cold-sensitive due to impaired heat shock factor 1 (HSF1) signaling [219] while *Bmal1* knockout mice show no difference in cold-stimulation responses compare to controls. An interesting study has demonstrated that increased light exposure (16 h per day) *vs.* standard light regimes (12 h light per day) leads to increased adiposity with no effects on food intake or locomotor activity. Such response is associated with decreased sympathetic input to BAT and reduced FFA uptake [220]. It is still elusive which players that regulate the proper functioning of the circadian clock machinery in BAT. For instance, TRP channels, in particularly TRPV1 and TRPM8, have been shown to affect the molecular clock since in *Trpv1* and *Trpm8* knockout mice, oscillatory profiles of clock genes are lost or disrupted [89, 90]. Moreover, β-adrenergic receptors are necessary for basal and cold-induced thermogenic responses, but are dispensable for molecular clock function [221]

**The endocrine pancreas** is a complex organ comprised of different cell types that regulate glucose homeostasis in a daily basis. There are two cell types of major importance in this process: insulin-secreting β-cells and the glucagon-secreting α-cells. It is widely established that insulin and glucagon levels are circadian independent of feeding regimes [222, 223]. Initial studies suggested an important role of the SCN in regulating daily rhythms of insulin and glucagon. However, such experiments were based on surgical SCN lesions, whose limitations are highlighted in "Organization of the circadian clock network". Several studies have demonstrated a functional circadian clock in pancreas responsible for controlling insulin secretion in a daily fashion in different mammalian models [224–226] including humans [227]. Amongst the clock genes, an interesting role has been suggested for *Dbp* and Thyrotroph Embryonic Factor (*Tef)* in pancreatic clocks [228, 229]. Omics approaches have shown that approximately 30% of the ß-cell transcriptome show a circadian oscillation and were involved in insulin-related vesicle trafficking and membrane fusion processes. Remarkably, transcriptomic data of ß-cell-specific *Bmal1* knockout mice show rhythm blunting for genes involved in exocytosis [230]. In vivo and in vitro transcriptomic analyzes of α- and β-cells reveal distinguished gene programs throughout the day in each cell type, which are believed to contribute to the temporal orchestration of insulin and glucagon release [231]. Neonatal pancreatic clocks are not fully functional at early stages and miRNAs were suggested to participate as regulators of emerging circadian clock functioning in the post-neonatal phase [232]. Global *Clock* deletion results in increased glucose levels and impaired glucose tolerance. Such effects are absent in young (3 months old) mice, but become evident in older (8 months old) animals [224]. Ex vivo stimulation with glucose of isolated pancreatic islets shows a marked reduction in insulin secretion in both young and old *Clock* knockout mice [224]. The global absence of *Bmal1* also yields impaired glucose-induced insulin responses [224]. Remarkably, a pancreas-specific *Bmal1* knockout results in elevated glucose levels, impaired glucose tolerance, and decreased insulin secretion already at a very young (2–4 months) age [224, 233]. The role of *Rev-erbα* has also been explored. Glucose-induced insulin secretion and β-cell proliferation are decreased when *Rev-erbα* is silenced [234]. Moreover, a ß-cell-specific *Bmal1* knockout results in hyperglycemia and impaired glucose tolerance [230, 235, 236]. Excitingly, *Bmal1* has been suggested to have an antioxidant role since in its absence ROS levels accumulate in the pancreas. In addition, in vitro administration of antioxidant may rescue glucose-induced insulin release via a NRF2-dependent pathway [235]. In summary, these data place the molecular clock as an important regulator of pancreas physiology. In humans, rhythmic clock gene expression has been described [227] and basal and stimulated insulin secretion is dependent on *CLOCK* [237]. Pancreatic islets from type-2 diabetic patients shown decreased clock gene expression rhythms which are correlated with insulin content and glycated hemoglobin [238]. For an in-depth view of the role of the molecular clock in the pancreas the reader is referred to excellent reviews [239, 240].

In addition to its canonical function as a nutrient absorptive organ,** the intestinal tract **plays a systemic regulating role due to its interaction the gut microbiome. An increasing body of evidence shows that gut microbiota influences immune responses, intestinal homeostasis, nutrient processing, and pathogen resistance. Many fecal bacteria show rhythms of abundance in the gut. In the absence of *Bmal1*, these rhythms are largely lost in both genders, but sex-related differences were also reported [241]. Not surprisingly, microbiome disbalance has been associated with increased risks of developing obesity, inflammatory diseases, cancer, diabetes, and psychiatric, respiratory, and metabolic disorders (reviewed in [242, 243]. Concerning intestinal physiological processes, several important parameters such as gastrointestinal motility and emptying [244–246], epithelial cell renewal [247–249], and absorption of nutrient and electrolytes display oscillatory profiles [250–252]. The presence of a local molecular clock has been confirmed in several parts of the intestine in mice and humans [252–254]. Strikingly, circadian *Nfil3* expression is affected by microbiota, STAT3 (signal transducer and activator of transcription 3), and the epithelial cell circadian clock. Remarkably, NFIL3 affects lipid uptake and storage, and modulates lipid absorption and export, thus placing NFIL3 as an important link between microbiota, molecular clocks, and host metabolism, and therefore, as a putative therapeutic target [255]. Gene knockout models have demonstrated the importance of the circadian clock in intestinal physiology. *Clock* knockout leads to the absence of circadian patterns of enterocyte activity and loss of food-induced synchronization [252, 256]. Interestingly, rhythmic calcium serum levels have been reported in mice and are lost in mice with targeted *Bmal1* deletion in the intestines due to disrupted calcium absorption, which consequently leads to increased calcium bone absorption. This process is dependent on a vitamin D receptor-related pathway [257]. For in-depth descriptions about the role of the molecular clock in intestinal regulation, please refer to recent reviews [250, 258].

**The liver** is the largest metabolic organ and responsible for a myriad of survival-limiting biological processes, of which we here highlight metabolism of nutrients and bile acids, xenobiotic metabolization, and synthesis of plasma proteins and cholesterol. As an object of study in the early years of molecular chronobiology of peripheral tissues [94], the liver has been one of the best-studied tissues in the circadian field. Reports on circadian oscillations in physiological aspects of the liver date back to the early 1960’s when rhythmic enzyme activity of pyruvate kinase, fatty acid synthetase, and glycogen-related enzymes were first described [259–261]. Data from the beginning of the century using circadian microarray analyses estimated that about 10% of all genes are rhythmically expressed in liver [262–264]. Recent transcriptomic techniques such as nascent RNA-seq, ChIP-seq, and proteomics, however, report a lack of overlap between rhythmic targets evaluated by each technique when compared to transcriptomics [28, 138, 140, 146], thus implicating post-translational modifications as an important circadian regulatory process Furthermore, circadian oscillations in gene programs associated with metabolism of carbohydrate, lipids, amino acids and bile acid, detoxification, and synthesis of plasma proteins and cholesterol have been documented [36, 262, 263, 265]. Experimental data from global or liver-specific clock gene deletion mice have revealed an important regulatory role of this machinery in both local (hepatocyte) but also systemic homeostasis, as described below. *Cry1* overexpression in leptin receptor mutant (*db/db*) mice was shown to improve insulin sensitivity [24]. *Clock* knockout mice show reduced plasma levels of insulin and increased circulating cholesterol, glucose, leptin, and triglycerides levels compared to wild-type controls, and consequently, are more prone to develop metabolic syndrome under a HFD regime [224, 266]. Moreover, global *Clock* knockout mice show a deregulated metabolic profile mainly related to pyrimidine salvage, lipid metabolism, and Krebs cycle-related pathways [267].

Liver-targeted *Bmal1* deletion in the liver leads to arrhythmic gene expression of glucose-related genes, increased glucose clearance, and hypoglycemia restricted to the fasting phase [268]. As expected, such responses do not perfectly correlate with the findings of global *Bmal1* knockouts in glucose metabolism, but corroborates the regulatory role of the molecular clock in the physiology of the liver [269]. Dual *Rev-erbα/β* knockout specifically in the liver results in loss of rhythmicity in almost of 90% of wild-type rhythmic transcripts, altered locomotor activity, increased circulating glucose and triglyceride levels, and a reduction in FFAs compared to controls [270]. Such deleterious effects of dual liver-specific gene knockout have been supported by an independent study [271]. Targeted *Rev-erbα* knockout in the embryonic stage or in adults, on the contrary, leads to only modest alterations in liver physiology [270, 272]. Recently, *Rev-erbα/β* knockout in the liver was shown to disrupt transcriptional and *de-novo* lipogenesis rhythms. Interestingly, hepatocyte-targeted deletion of *Rev-erbα/β* also affects the transcriptome and metabolome of non-hepatocyte cells within the liver. Moreover, in livers of control mice, submitted to time-restricted food access, transcriptomics reveal a phase shift of 12 h in gene expression, which is completely lost in *Rev-erbα/β* knockouts [273]. On the other hand, liver-specific *Rorγ* knockout mice show daytime improved insulin sensitivity and glucose responses as result of decreased hepatic gluconeogenesis. In addition, bioinformatic analyses revealed that RORγ is a regulatory player of several glucose-related genes by interacting with *RORE*s in their promoter regions [274]. In another study, liver-specific *Rorγ* knockout mice show reduced lipid levels in liver and blood as well as reduced bile acid synthesis [275]. In contrast of the beneficial effects of *Rorγ*, as described above, the removal of *Rorα* in the liver results in increased HFD-induced hepatic steatosis through AMPK and liver X receptor α (LXRα)-dependent pathways [276]. Remarkably, circadian lipidomic approaches demonstrate that in absence of *Per1* and *Per2*, a similar fraction (~ 20%) of all lipids continue to display an oscillation. However, lipid composition and phasing are altered in *Per1*/*2* knockout mice fed ad libitum. Such findings demonstrate that, even in the absence of a functional transcriptional circadian clock mechanism, rhythms in lipids may still persist [277]. Food restriction regimes protect against the development of metabolic syndrome in mice [278] in a clock-dependent way [279] as well as in a functional peripheral clock network only in LD, but in a SCN-independent manner [280], thus following the federated model of circadian regulation (See Sect. 1.2). Recent evidence supports an interesting regulatory role of neutrophils in liver rhythms. Reduced neutrophil infiltration, under different experimental conditions, is protective against jetlag and diet-induced liver steatosis in a FGF21-dependent manner [281]. Sirtuin1 (SIRT1) is an important nutrient-sensing protein that interacts with the molecular clock. It upregulates the expression of *Nicotinamide phosphoribosyltransferase* (*Nampt*) via* E-box* interaction. Interestingly, NAMPT is responsible for daily oscillations in intracellular NAD^+^ levels, which are used by SIRT1 as a cofactor to deacetylate clock- and energy metabolism-related proteins [282, 283]. BMAL1/CLOCK heterodimers bind to *Sirt1* promoter leading to its transcription [284]. SIRT1-dependent deacetylation contributes to the rhythmic expression of its target genes to modulate liver physiological processes [282–285]. SIRT7 deacetylates CRY1, leading to its degradation, as well as regulating liver molecular clock and glucose homeostasis [286].

An important feature of liver is its metabolism of xenobiotics. It has been shown that lipophilic drugs are more rapidly absorbed in the morning compared to the evening in humans while no time-of-day dependence in hydrophilic drug absorption has been reported (reviewed in [287]). Higher morning gene expression of some organic anion transporting polypeptides (OATPs), organic anion transporters (OATs), and organic cation transporters (OCTs) compared to the evening was reported in mice [288]. Phase-I xenobiotic metabolism mainly consists of oxidation, reduction, and hydrolysis, processes that are mainly carried out by cytochrome p450 enzymes. Oscillation at the mRNA levels in several members of the p450 superfamily has been reported with peaks during the dark phase [288]. Interestingly, loss of positive transcriptional factors (*Bmal1 or Clock*) or negative members (*Pers*) of the clock TTFL leads to low or high xenobiotic metabolism, respectively [289]. In phase II, detoxification takes place via conjugation to charged compounds such as glutathione, sulfate, and others, thus making a compound more polar and easier to be transported. Gene expression of phase-II-related enzymes has been reported to be abundant during different times within the light phase (peak in the early day or around the day-night transition) [288]. Lastly, phase III comprises several types of membrane transporters of the multidrug resistant protein (MRP) family. Circadian rhythms in this class are less marked, but some transporters show an oscillatory profile, peaking during the light phase in a similar fashion as phase-II genes [288]. Overall, it is accepted that the abundance of phase-I enzyme-related transcripts increases during the dark phase while phase-II and -III transcripts are more abundant during the light phase [288]. More in-depth reviews are found here [290, 291].

## Conclusions

The circadian clock system is a complex and pervasive network that directly or indirectly controls a significant portion of all biological processes. Its role as a systemic regulator is seen in experimental models of gene silencing or knockout. Advancements in tissue-specific clock gene modulation has been important for a better understanding of tissue clock gene organization at both cellular as well as systemic levels. In addition to its molecular basis, the organizational aspects of the circadian clock network have received increased attention over the last years. Understanding how this biological timing machinery works at all levels of organization will be important to fully assess the consequences of circadian rhythm disruption by modern lifestyles and devise ways to prevent chronodisruption and treat associated diseases and conditions.
